# Protective Effect of *Thunbergia laurifolia* (Linn.) on Lead Induced Acetylcholinesterase Dysfunction and Cognitive Impairment in Mice

**DOI:** 10.1155/2013/186098

**Published:** 2013-12-21

**Authors:** Moe Pwint Phyu, Jitbanjong Tangpong

**Affiliations:** ^1^Biomedical Science, School of Allied Health Sciences and Public Health, Walailak University, Nakhon Si Thammarat 80160, Thailand; ^2^School of Allied Health Sciences and Public Health, School of Allied Health Sciences and Public Health, Walailak University, Nakhon Si Thammarat 80160, Thailand

## Abstract

*Thunbergia laurifolia* (linn., TL), a natural phenolic compound, has been reported to have many benefits and medicinal properties. The current study ascertains the total phenolic content present in TL aqueous leaf extract and also examines the antioxidant ability of the extract in preserving acetylcholinesterase (AChE) activity of mice exposed to lead *in vivo* and *in vitro* model. Mice were given lead acetate (Pb) in drinking water (1 g/L) together with TL 100 and 200 mg/kg/day. The result showed that Pb induced AChE dysfunction in both *in vitro* and *in vivo* studies. TL significantly prevented Pb induced neurotoxicity in a dose-dependent manner which was indicated by comparatively better performance of TL treated mice in Morris Water Maze Swimming Test and increased AChE activity in the tissue sample collected from the brains of these mice. TL also exhibited the greatest amount of phenolic content, which has a significant positive correlation with its antioxidant capacity (*P* < 0.05). Taken together, these data suggested that the total phenolic compounds in TL could exhibit antioxidant and in part neuroprotective properties. It may play a potential treatment strategy for Pb contamination.

## 1. Introduction

Lead (Pb) is a ubiquitous environment and industrial pollutant that mainly conveyed to humans through water, food, and occupational sources. Lead can also be transmitted through maternal milk [1, 2], and even low levels of lead (0.3%) exposure can cause long-lasting cognitive deficits [3]. Furthermore, removal of lead exposure at weaning still produced learning deficits in adult rats [4]. Lead is a highly neurotoxic agent that causes functional and structural abnormalities in the brains. Pb poisoning is a medical condition that is toxic not only to brain but also to many other organs and tissues including heart, bones, intestines, kidneys and reproductive organs, for example reproductive organs in female: the internal genital structures of the female include the ovaries, Fallopian tubes, uterus (womb) and vagina. The nervous system is the primary target for the Pb exposure and the developing brain appears to be especially vulnerable to Pb neurotoxicity [5, 6].

Acetylcholinesterase (AChE) or acetylhydrolase is a serine protease that hydrolyzes the neurotransmitter acetylcholine to be acetyl CoA and choline. AChE is found at mainly neuromuscular junctions and cholinergic brain synapses, where its activity serves to terminate synaptic transmission. Other studies have shown that the deleterious effect of Pb exposure on memory could be related to its capacity to induce cholinergic dysfunction in brain [7, 8]. Since cholinergic system is responsible for the behavioral manifestations in animals, observation of Pb induced impairments in AChE system can be attributed to cognitive dysfunction [7].

Although chelating therapy is currently an available treatment of Pb neurotoxicity, it is observed to have many adverse effects such as divalent metal ion imbalance and it is also ineffective in improving previous nerve injury induced by Pb. Currently, no efficient drugs are available for treating chronic lead induced cognitive deficits [9, 10]. The trumpet vine *Thunbergia laurifolia* (Linn.) (TL) is a Thai medicinal plant known for its antimutagenic, anti-inflammatory, and antipyretic properties [11–13]. Aqueous extract preparation of fresh leaves, dried leaves, dried root, and bark of TL has been used in detoxification and first aid treatment for poisoning from insecticides, ethyl alcohol, arsenic, and strychnine [14]. The phenolic compound of TL leaf extracts could function as superior antioxidants and as well as a chelating agent. Tangpong and Satarug stated that TL leaf extract (200 mg/kg body weight) thus reduced neuronal cell death and memory loss caused by Pb uptake in mice, and the antioxidant activities of the TL leaf extract might account for these effects [15].

In the present study, we determined the total phenolic content and antioxidant capacity present in TL aqueous leaf extract and whether TL can protect Pb induced neurotoxicity, learning deficit, and memory loss by altering AChE activity *in vitro* and *in vivo*.

## 2. Materials and Methods

### 2.1. Chemicals and Preparation of *Thunbergia laurifolia* (Linn.) Aqueous Leaf Extract

Lead acetate and all chemicals were purchased from Sigma-Aldrich, St. Louis, MO, USA. TL leaves were collected during April-May at Nakhon Si Thammarat, Thailand. Leaves were air dried and ground in a blender to a fine powder. TL leaf powder, 100 g, was extracted with 1000 mL of boiling water for 15 min. The TL leaf aqueous extracts were filtered by Whatman no. 1 and then lyophilized using freeze dryer at −20°C for 20 h (Eyela, Tokyo, Japan). The powder was stored at 20°C until used.

### 2.2. Animals

Forty-two male ICR mice (30–33 g), eight week-old, were divided into seven groups of six mice each and maintained on 12 h light/dark cycle in a temperature controlled (23 ± 2°C) room. The mice had free access of food and water. All animal experimental procedures were approved by the Animal Care and Use Committee of Walailak University.

### 2.3. Animal Treatment

Mice were treated with lead acetate Pb(CH_3_COO)_2_·3H_2_O in drinking water at 1 g/L. TL at dose of 100 and 200 mg/kg and vitamin E 100 mg/kg were orally administered at 7.00–8.00 am once a day. Mice body weight were measured before and after treatment and were euthanized at 8 weeks after treatment. Mice were randomly divided into 7 groups with 6 mice in each group. Group 1 served as a control and mice in this group received sodium acetate as CH_3_COONa·3H_2_O in the same molar concentration as those in group 2. Group 2 was Pb only and received Pb, as lead acetate Pb(CH_3_COO)_2_·3H_2_O in drinking water at 1 g of Pb/L. Group 3 received once a day TL extract at 200 mg/kg body weight via tube gavages. Group 4 received Pb in drinking water together with TL extract at 100 mg/kg body weight once a day via tube gavages. Group 5 received Pb in drinking water with TL extract at 200 mg/kg body weight once a day. Group 6 received Pb in drinking water (1 g Pb/L) with vitamin E at 100 mg/kg body weight once a day via tube gavages. Group 7 served as vitamin E control and mice in this group received vitamin E vehicle (vegetable oil).

### 2.4. Sample Collection

Animals were anesthetized using Nembutal/sodium (65 mg/kg) and blood was obtained via left ventricle puncture and perfusion was followed with the use of cold phosphate buffer saline, pH 7.4. The brain was removed and homogenized in cold PBS, containing a mixture of protease inhibitors (leupeptin, pepstatin, and aprotinin) prior to centrifugation at 15,000 ×g for 15 min. The resultant supernatant was collected for further analysis.

### 2.5. Total Phenolic Content and Total Antioxidant Capacity of TL Aqueous Leaf Extract

The total phenolic content of the extracts was measured according to the Folin-Ciocalteu method described by the method of [16] as modified. The concentrations of phenolic content in TL extracts were expressed as gallic acid equivalents (GAEs). Briefly, 12.5 *μ*L of extract of different concentrations (0.1, 0.25, 0.5, 1 mg/mL) and control (distilled water was used instead of extract) were added in the 96-well microplate. Then 12.5 *μ*L of Folin-Ciocalteu's phenol reagent was added to each well. After 5 min, 125 *μ*L of saturated sodium carbonate (Na_2_CO_3_) solution (~7.5%) was added to the mixture. The reaction mixtures were incubated at room temperature for 30 min. DW was used as blank. All assays were conducted in triplicate. The absorbance was determined at 765 nm with a microplate reader. Gallic acid solutions with concentrations ranging from 0 to 100 mg/L were used for calibration. A dose response linear regression was generated by using the gallic acid standard absorbance and the levels in the samples were expressed as gallic acid equivalents (mg of GAEs/mg dw).

2,2′-azino-bis(3-ethylbenzothiazoline-6-sulphonic acid) or ABTS also forms a relatively stable free radical, which decolorizes in its nonradical form. The analysis of ABTS^∙+^ radical cation scavenging activity was determined according to the method of Rice-Evans et al. [17]. In this method, an antioxidant is added to a pre-formed ABTS radical solution and after a fixed time period the remaining ABTS^∙+^ is quantified microplate reader at 734 nm. ABTS^∙+^ was produced by reacting 7 mM ABTS in H_2_O with 4.9 mM potassium persulfate (K_2_S_2_O_8_), stored in the dark at room temperature for 12–18 h. The ABTS^∙+^ solution was diluted to give an absorbance of 0.750 ± 0.025 at 734 nm. Then 180 *μ*L of ABTS^∙+^ solution was added to 20 *μ*L of TL solution in distilled water at different concentrations (0.1–1 mg/mL). The absorbance was recorded 3 min after mixing and the percentage of radical scavenging was calculated for each concentration relative to a blank containing no scavenger. The extent of decolorization is calculated as percentage reduction of absorbance. For preparation of a standard curve, different concentrations of Trolox were used and expressed in millimole of Trolox equivalent per gram of dry weight (mM TEAC/g dw).

The correlation coefficient (*R* ^2^) of total antioxidant capacity and total phenolic content of TL aqueous leaf extract were considered.

### 2.6. Determination of Acetylcholinesterase Activity

The activity of AChE *in vitro* and *in vivo* studies were determined by cholinesterase (CHE) from BioSystems S.A. Costa Brava, 30, 08030 Barcelona, Spain,s and expressed as U/L. The specific activity of AChE was determined as described by Ellman et al. [18]. The reaction mixture contained 3 mL of 0.1 M phosphate buffer (pH 8), 20 *μ*L of 0.075 M acetylcholine iodide, and 100 *μ*L of 0.01 M 5, 5-dithiobis-2-nitrobenzoic acid. The reaction was initiated with the addition of 100 *μ*L of brain homogenate sample. The contents were incubated for 30 min at room temperature and the color absorbance was measured at 412 nm in spectrophotometer (Hitachi, Model U-2000). The enzyme activity was expressed as U/L *in vitro* study and U/mg protein in brain homogenate tissues.

### 2.7. Water Maze Swimming Test

The learning and memory test followed the method of Morris [19] as described by Kim et al. [20]. Briefly, animals were tested in a circular pool of 190 cm in diameter and 45 cm in height with a featureless inner surface. The pool was filled with water to the depth of 30 cm. The pool temperature was maintained at 25 ± 1°C. The pool was divided into four quadrants of equal area in each quadrant. A clear plastic platform, 6 cm in diameter and 29 cm in height, was placed in one of the pool quadrants. The first experimental day was for training for 120 s without a submerged platform. On the following 5 days, the mice were given two daily trials with an intertrial interval of 30 min in the presence of the platform. When a mouse located the platform, it was permitted to remain on it for 10 s. If the mouse did not locate the platform within 120 s, it was placed on the platform for 10 s. The animal was taken to its cage and blotted dry with Kimwipe papers. For each trial session, the time a mouse took to find a hidden platform, termed latency, was recorded. The latency time data of a final trial were recorded.

### 2.8. Statistical Analysis

Data were expressed as mean ± SEM. The data obtained was analyzed using the Student *t*-test and one way ANOVA which need to compare treatment groups. The *P* values of ≤0.05 were considered to identify statistically significant levels.

## 3. Results

### 3.1. Total Phenolic Content and Antioxidant Activity

**Figure 1:**
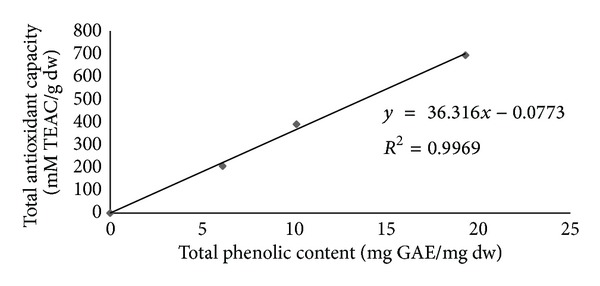
Linear correlation of Trolox equivalent antioxidant capacity (mM TEAC/g dw, *Y*) versus the total phenolic content (mg GAE/mg dw, *X*) of *Thunbergia laurifolia* (Linn.) aqueous leaf extract.

Table 1 showed that TL aqueous leaves extract (0, 0.05, 0.1, 0.2 mg/mL) contains total phenolic content and total antioxidant capacity. The correlation between the antioxidant activity and total phenolic content was determined. The antioxidant capacity of extract appears to be largely influenced by the content of total phenolic compound (*R* ^2^ = 0.996) (Figure 1).

### 3.2. *In Vitro* Effects of *Thunbergia laurifolia* (Linn.) against Lead Induced Acetylcholinesterase Activity

**Figure 2:**
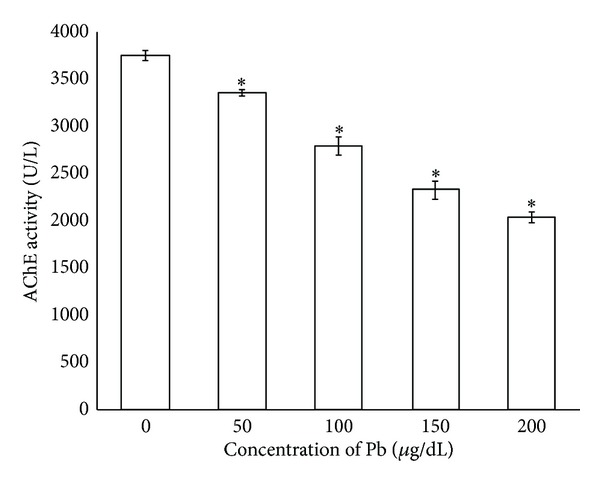
Lead inhibited acetylcholinesterase activity *in vitro* study. Data are presented as mean ± SEM. *P < 0.05 compared between Pb exposure group and untreated control group.

**Figure 3:**
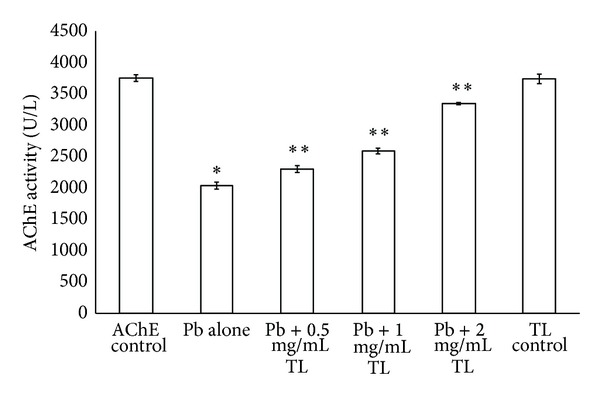
The protective effect of *Thunbergia laurifolia* (Linn.) against Pb inhibited acetylcholinesterase activity *in vitro* study. Each value is expressed as mean ± SEM. *P < 0.05, compared with the control group; **P < 0.05, compared with Pb alone treated group.

In order to determine whether TL can attenuate the neurotransmitter system damage, we measured the activities of AChE. According to Figure 2, lead at higher concentration tested *in vitro* (50 to 200 *μ*g/dL) promoted significant decrease on AChE activity (3,356 ± 34.4 to 2,037 ± 56.9 U/L), respectively. Interestingly, the treatment with TL, on the other hand, restored the activities of AChE (*P* < 0.05) (Figure 3). There was no significant difference in activities of AChE between the control group and TL alone treated group.

### 3.3. *Thunbergia laurifolia* (Linn.) Aqueous Leaf Extract Protect Acetylcholinesterase Activity on Lead Treated Mice Brain

**Figure 4:**
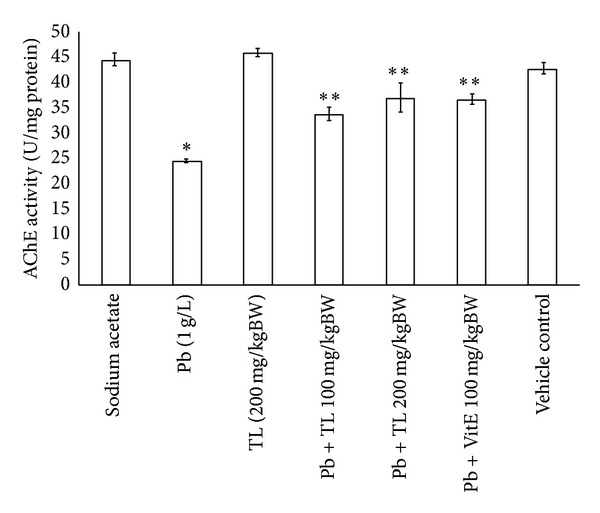
The protective effect of *Thunbergia laurifolia *(Linn.) against Pb inhibited acetylcholinesterase activity of brains tissues in mice model. Each value is expressed as mean ± SEM. *P < 0.05, compared with the untreated control group; **P < 0.05, versus Pb treated group.

As shown in Figure 4, the activity of AChE was markedly decreased in the brains of Pb treated mice as compared with the untreated controls (*P* < 0.05). However, there was significant improvment in the activities of AChE in cotreatment of Pb with TL aqueous leaf extract at 100 mg/kg or 200 mg/kg and vitamin E control group (*P* < 0.05).

### 3.4. Latency Time in Pb Exposure Mice

**Figure 5:**
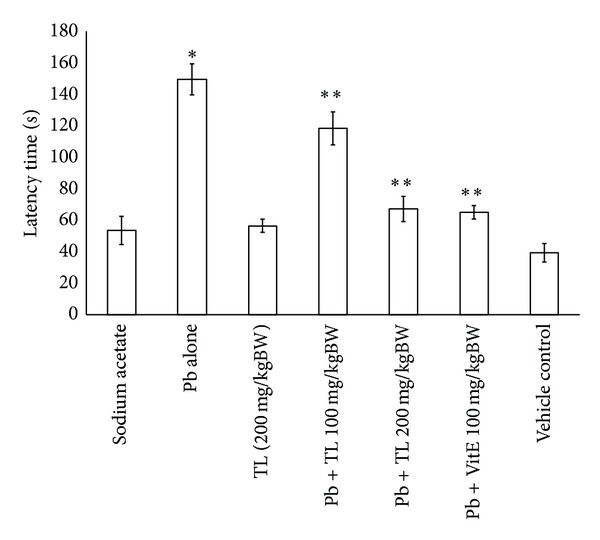
*Thunbergia laurifolia *(Linn., TL) aqueous leaf extract on latency time (seconds) in Pb treated mice using Water Maze Swimming Test. Data are presented as mean ± SEM. *P < 0.05, compared with the control group; **P < 0.05, compared with Pb alone treated group.

Figure 5 presents effects of treatments on Water Maze Swimming Test results. Average latency time is defined as time in seconds taken to locate a platform. At the first day of trial, there were no significant differences in latency time observed among animal groups (data not shown). At fifth day of trial, an average latency was markedly increased in the group treated with lead only, compared with those groups of control group or mice receiving lead plus vitamin E or lead plus TL leaf extract at 100 mg/kg or 200 mg/kg body weight (*P* < 0.05). Treatment with TL alone showed no effects on latency time.

## 4. Discussion

This study evaluated the effects of TL on the impairment of memory and decreased AChE activity induced by lead. We also found that the total phenolic content and total antioxidant content are high in the TL aqueous leaf extract (Table 1). It is well known that phenolic compounds contribute to quality and nutritional value in terms of modifying color, taste, aroma, and flavor and also in providing health benefits effects. They also serve in plant defense mechanisms to counteract reactive oxygen species in order to survive, prevent molecular damage, and disruption by microorganisms, insect and herbivores [21]. However, the correlation between total phenolic content and antioxidant activity is not clear. Our results show that the correlation between total phenolic content and antioxidant activity of TL aqueous leaf extract had correlation coefficient of *R* ^2^ = 0.996 (Figure 1). This suggests that the antioxidant capacity of TL aqueous leaf extract results from the contribution of phenolic compounds. They may also have metal chelating potential [22].

The nervous system is the primary target for the Pb exposure [5]. A number of studies have reported that Pb exposure can decrease AChE coupled with increased Acetylcholine [5, 23]. Pb may have high affinity with free SH group of this enzymes proteins and its binding can alter their function [24, 25]. This may be the reason for the observed inhibition of AChE activity (Figure 2) in both *in vitro* and *in vivo* mouse brain tissues which consistent with the earlier reports [26, 27].

*Thunbergia laurifolia* (Linn.) is one of medicinal plant enrich phenolic compound which is a potential metal chelator. Efficacy of TL against Pb inhibited acetylcholinesterase activity has been shown in *in vitro* studies (Figure 3). TL appears to be a suitable antidote for Pb poisoning in mice. The previous study in our group also showed that TL markedly decreased the lead levels, which may explain the neuroprotective effect of TL [15].

At cholinergic synapses, AChE plays a crucial role in synaptic transmission by controlling the action of choline disorders of AChE that has been implicated in a variety of neuropsychiatric disorder [22]. However, TL significantly restored AChE of brain in Pb treated mice, suggesting that TL could protect neurotoxicity by reversing the Pb induced AChE dysfunction (Figure 4).

Pb is a prevalent occupational and environmental neurotoxicant which also causes cognitive and behavioral defects [28]. Exposure to Pb is associated with neurobehavioral and psychological alterations, including the inhibition of long term potentiation (LTP), growth retardation and learning and memory impairment [29]. In this study, mice exposed to Pb also presented memory loss, as observed by using the Morris Water Maze Swimming Test which showed significantly longer latency time than did control animals to find hidden platform during the entire period of acquisition (Figure 5), consistent with previously reported findings [4].

In conclusion, this study suggests that high phenolic content and antioxidant activity of TL have potent protective effects against Pb induced AChE dysfunction. Our data showed that TL prevented Pb induced memory impairment and AChE dysfunction in a dose-dependent manner, indicated by the *in vitro* and *in vivo* studies. In coexposure experiment, TL extract, and vitamin E similarly alleviate symptoms of lead poisoning. TL extract may play the other function role as Pb chelating agent and it is water soluble thus high level of TL can be tolerated and excreted. Thus, this study indicates that dietary supplement TL may have benefits to alleviate the learning deficit and memory loss due to lead exposure.

## Figures and Tables

**Table 1:** Total phenolic content and total antioxidant capacity of *Thunbergia laurifolia* (Linn.) aqueous leaf extract.

Concentration of *Thunbergia laurifolia* (Linn.) (mg/mL)	Total phenolic content (mg GAE/mg dw)	Total antioxidant capacity (mM TEAC/g dw)
0.05	6.11 ± 0.59	206 ± 11.84
0.1	10.12 ± 0.34	390 ± 12.46
0.2	19.3 ± 1.24	694 ± 11.84

Data are presented as mean ± SEM, dw: dry weight.
